# Phylogenetic analysis of hepatitis C virus among HIV/ HCV co-infected patients in Nigeria

**DOI:** 10.1371/journal.pone.0210724

**Published:** 2019-02-06

**Authors:** Juliet A. Shenge, Georgina N. Odaibo, David O. Olaleye

**Affiliations:** Department of Virology, College of Medicine, University of Ibadan, Ibadan, Nigeria; University of Cincinnati College of Medicine, UNITED STATES

## Abstract

Hepatitis C virus (HCV) infection has been associated with liver disease including liver cirrhosis and hepatocellular carcinoma (HCC) in chronically-infected persons. However, in HIV/HCV co-infected patients, increased rate of progression to cirrhosis and HCC has been reported. Limited information exists regarding genetic variants of HCV circulating among co-infected patients, which could be important in the design of broadly protective vaccine and management of the disease. Here, we determined the genotypes of HCV isolates circulating among HIV/HCV co-infected patients in Ibadan, southwestern Nigeria. One hundred and twenty-five HIV/HCV IgM positive samples obtained from HIV laboratory, University of Ibadan were used for this study. HCV NS5B gene was amplified using polymerase chain reaction (PCR). The amplified NS5B gene was sequenced using gene specific primers. Twenty isolates were amplified, out of which 13 were successfully sequenced. Phylogenetic analysis of the 13 sequenced isolates showed three HCV subtypes 1a, 3a and 5a belonging to genotypes 1, 3 and 5 respectively. Ten isolates (77%) belong to subtype 5a, followed by 2 isolates (15%) subtype 1a and 1 isolate (8%) was subtype 3a. The predominant HCV genotype was 5, followed by genotype 1 (subtype 1a). The findings, as well as the observed mutations in NS5B gene, indicate the need for screening and monitoring of HIV/HCV co-infected patients. Further study to determine the phylogeny of isolates circulating in other parts of Nigeria will be carried out.

## Introduction

Hepatitis C virus (HCV) still affects more than 185 million people worldwide despite availability of highly effective antiviral therapy such as direct acting antiviral agents (DAA) [1]. According to [2], an estimated 3–4 million people become infected every year, representing more than three percent of the world’s population that are chronically infected, with most of these cases occurring in Africa [2, 3]. It is also estimated that about 350, 000 people die from liver failure and liver cancer caused by hepatitis C disease each year. Furthermore, about 2.3 million people infected with HIV are actually co-infected with hepatitis C virus globally [4].

It has been reported that about one third of HIV-infected patients are also infected with HCV with an undesirable impact on HCV pathogenesis [5]. HIV destroys the immune system by depleting CD4-bearing T cells while HCV causes necrosis of the hepatocytes. Liver disease progression is known to be influenced by some viral and host factors, which include viral load, viral genotype, period of infection, gender, age, alcohol consumption as well as co-infection with hepatitis B virus (HBV) or Human Immunodeficiency Virus (HIV) [6].

Studies have also shown that about 75% of HCV related deaths occur among adults ages 45 to 65 [7] and mostly among individuals having co-infections with HIV, HBV and other liver complications such as cirrhosis and hepatocellular carcinoma [8, 9]. Chronic infection with HCV has been reported as one of the causes of chronic liver disease, the reason for most Orthotopic Liver Transplantation (OLT) in the USA [10]. In sub-Saharan Africa, HBV is the main cause of hepatocellular carcinoma (HCC), followed by HCV mostly as a result of chronic hepatitis with complications from liver cirrhosis [11], especially in HIV co-infected patients [12]. The rate of fibrosis has been estimated to be 3 or more times higher in HIV/HCV co-infected patients, than in patients that are infected with HCV alone [13]. HIV is also known to accelerate the progression of liver disease in HCV-infected persons because HCV replication increases in the presence of HIV resulting in elevated liver HCV RNA levels [14, 15]. Detrimental effect of HCV on HIV infection includes a significant reduction of CD4 cells and total CD4 percent [16]. Independent of HIV or HCV infection, depletion of CD4 cell enhances progression to cirrhosis [17].

HCV is a member of the *Flaviviridae* family and the only member of the genus Hepacivirus. The virus is a small enveloped, spherical particle with a positive sense, single-stranded RNA genome. The genome consists of a single, open reading frame (ORF) that is 9600 nucleotide bases long and 2 untranslated but highly conserved regions namely; 5'-UTR and 3'-UTR located at both ends of the genome [18]. The genome encodes a single polyprotein, starting with the core proteins (structural) and ending with the NS5B proteins (non-structural proteins, which code for RNA polymerase [19].

The NS5B gene codes for RNA-dependent RNA polymerase (RdRp). It is essential for viral maturation and plays an important role during replication of the virus. The gene represents an ideal target for the development of antiviral drugs. Typically, phylogenetic analysis of the non-structural protein (NS5B) coding region is able to differentiate both genotypes and subtypes of the virus [20]. Due to the fact that the virus evolves rapidly, lack of proofreading activity of the RNA-dependent RNA polymerase results in extreme sequence variability resulting from frequent nucleotide substitutions and this represents marked genetic heterogeneity in HCV [21]. Accumulation of these nucleotide substitutions during HCV replication has resulted in the emergence of seven major HCV genotypes (1–7), each further divided into subtypes based on their genetic diversity [22, 23]. Numerous subtypes of HCV have been identified [24].

HCV genotypes are distributed differently throughout the world [25]. Differences also exist in the global distribution of its subtypes [26, 27]. Divergent strains of genotypes 1 and 2, have been found to be endemic in West African countries including; Burkina Faso, Ghana, Guinea Bissau, Benin Republic and Nigeria. Genotype 3 is found in south Asia, genotype 4 in central Africa and Middle East while genotype 6 is distributed in south-east Asia [27]. Genotype 5 has been found to be common in the northern region of South Africa and Belgium while genotype 7 has been reported only among the central African immigrants in Canada [28].

Limited studies exist concerning HCV genotypes distribution among co-infected persons. However, it has been reported that response to interferon and ribavirin-based regimens among HIV/HCV co-infected patients, depends largely on HCV genotype. It was observed that sustained virologic response is achieved faster in only HCV-infected patients, than in HIV/HCV co-infected patients. Notably, HCV genotype 2 or 3 responds faster to interferon and ribavirin, than HCV genotype 1, making response to antivirals slower in co-infected patients, than in HCV mono-infected patients [29]. Hence, treating HIV may slow progression of HCV-induced fibrosis by improving immune function and modulatory effects of interferon-based HCV treatment and thus improve response to HCV treatment and disease prognosis [30].

In Nigeria, there have been limited studies on the genetic diversity of HCV among various sub-populations [31]. Most of the previous studies from the country were mainly serology-based [32, 33]. Reports have shown that HBV and HCV infection accounts for large number of chronic liver disease and hepatocellular carcinoma (HCC) in Nigeria [34, 35], hence, the need for molecular studies that will provide information on some of the HCV epidemic determinants, types and risk factors. Information on the genetic variants of HCV in Africa is very crucial for better understanding of its epidemiology, pathogenesis, evolutionary dynamics and design of broadly-protective vaccine against infection with the virus [36].

## Materials and methods

### Study population and sample collection

This study was cross-sectional, in which blood samples of 125 HCV/HIV–co-infected individuals (68 males and 57 females, age range 3 months to 83 years) referred from different clinics at the University College Hospital, Ibadan to the Department of Virology of the same hospital for diagnosis were used. The samples were tested for IgM antibodies to HIV and HCV by the laboratory using ELISA. Positive samples (HCV/HIV) stored at -80°C were then retrieved for this study after approval by the ethical committee. Ethical approval for the study was received from the University of Ibadan/University College Hospital Ethical Committee under the assigned number UI/EC/ 14/0019, before commencement of study. The need for consent from individual participants was waived by the ethics committee.

### HCV RNA extraction and reverse transcription

RNA was extracted from aliquots of plasma sample, from which 5 ml blood was used according to manufacturer’s instructions of the commercially available extraction kit used (Jena Bioscience total RNA Purification kit, Germany). Reverse- transcription (first strand cDNA synthesis) of the extracted RNA was performed using random hexamer using Script cDNA synthesis kit (Jena Bioscience, Germany), in a final volume of 20 μl assay. The synthesis conditions were 42^o^ C for 10min followed by incubation at 50°C for 45min.

#### Amplification of NS5B gene

NS5B gene segment of the virus located at positions 8275–8616 was amplified using a nested PCR protocol with 5 μl of gene specific primers [26] and 2.5 μl of the RT-PCR product as the template. Amplified fragments were visualised using gel electrophoresis in 1.5% agarose gel concentration. Sequence of primers and the cycling conditions for the nested PCR are as follows: In the first round of PCR the primers used were NS5B-k1 for Forward: 5`TGGGGATCCCGTATGATACCCGCTGCTTTGA and NS5B-k2 reverse reaction: 5`GGCGGAATTCCTGGTCATAGCCTCCGTGAA. The cycling condition is 95°C for 5min, 94°C for 30sec, 50°C for 30sec, 72°C for 45sec,72^o^ C for 10min, for 30cycles. The expected size of amplification product for the first round PCR was 400bp. Five microlitre of the first round PCR product was used as the template for the second round PCR. The nested PCR was performed in a 25μl reaction mixture with 2.5 microlitres with forward primer- NS5B-122: CTCAACCGTCACTGAGAGAGACAT and reverse primers NS5B-R1- GCTCTCAGGCTCGCCGCGTCCTC. The thermal cycling condition was same as the first round reaction but for 45 cycles instead. The PCR product with the expected band size of 301bp was detected by electrophoresis in 1.5% agarose gel and visualized using Bio-Rad Gel Doc XR+ System.

#### DNA sequencing

The PCR products were purified with ExoSAP Amplicon Purification kit (Applied Biosystems, Foster city, CA,) according to manufacturer’s instructions and sequenced with ABI V3.1 Big dye terminator (Applied Biosystems, Foster city, CA). The second- round PCR products and the same inner primers used for the nested PCR were used for sequencing. Sequencing reactions were commercially carried out by INQABA BIOTEC, South Africa. Nucleotide sequences of the partial HCV NS5B gene obtained among HIV patients co-infected with HCV are available at the repository Doi: 10.6084/m9.figshare.7471454.

#### Editing of sequences

The electropherogram of each sequence was studied and edited using CLC Main Workbench 7.6.2 software (CLC Bio, Cambridge, MA, USA). Each consensus sequence generated was blasted in NCBI nucleotide blast to determine HCV reference sequences with closest matching identity or relatedness to study sequences.

#### Phylogenetic analysis

All sequences together with HCV reference prototype sequence H77 and GenBank sequences from different continents including Africa, North America, Europe and Australia, spanning 300-310nt of the NS5B gene {HCV H77 position, 8275–8616 (GenBank, NC.004102.1)} obtained from HCV sequence Database [27], were aligned using Clustal W 1.6 method in MEGA 7.0 version software [37]. The evolutionary history was inferred using the Neighbor-Joining method [38].

Viral diversity and the ancestral relationships among sequences and the reference sequences were determined by Neighbor-Joining phylogeny with Maximum Composite Likelihood model [38, 39]. Test of phylogeny, that is, the percentage replicate trees in which the associated taxa clustered together was performed with bootstrap replicates of 1,000 and branch support values of >60%.

Amino acid sequences were translated with the standard genetic code on MEGA7.0 [40]. Amino acid substitutions generated in this study were aligned with prototype H77 strain (GenBank accession number NC.004102.1).

## Results

**Fig 1:**
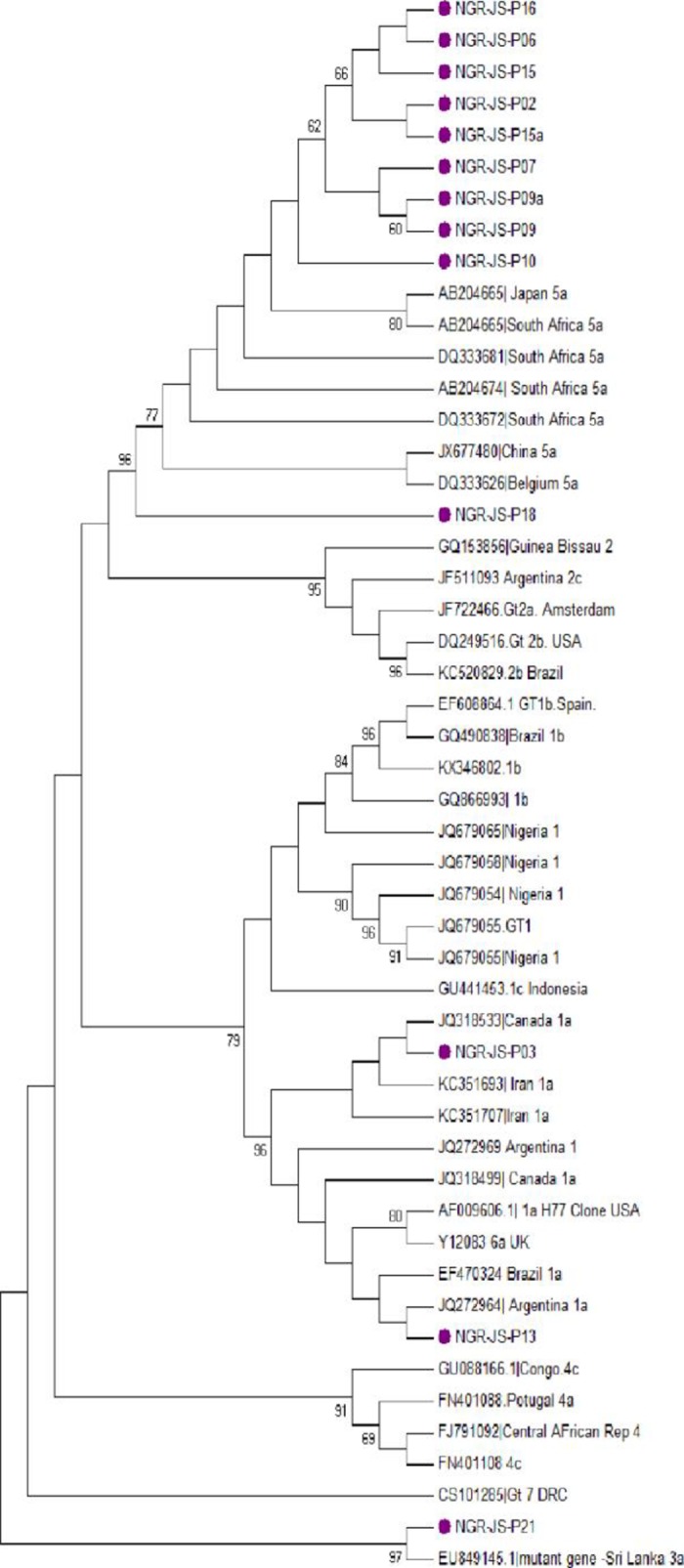
Phylogeny of HCV NS5B genes (blue blocks) in HCV patients co-infected with HIV. Tree was constructed using Neighbor-Joining method with Maximum Composite Likelihood model. Study sequences are marked with solid blocks showing genotypes 5a, 1a and 3a locations on the tree.

**Fig 2:**
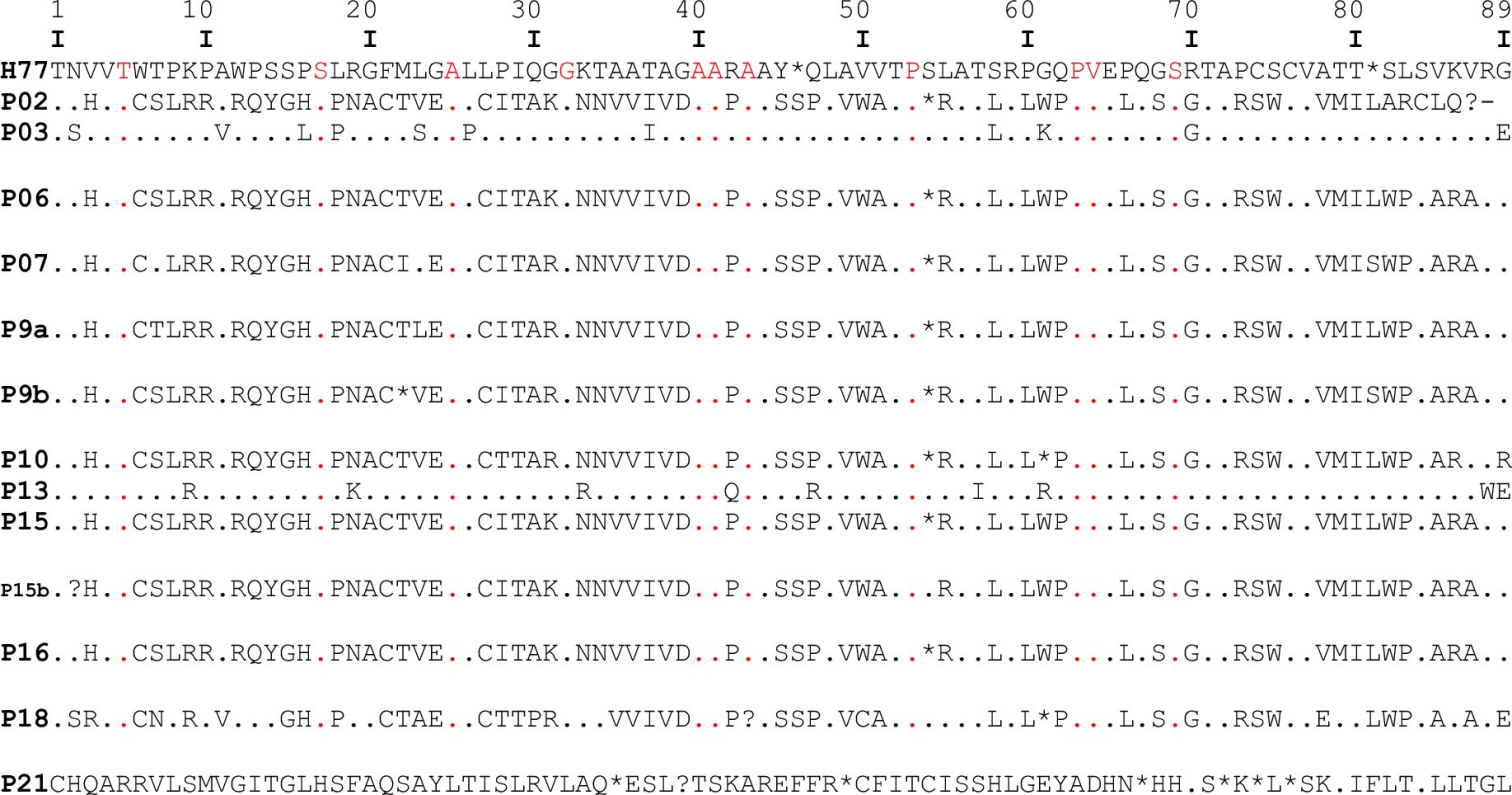
Amino acid alignment of all our HCV sequences in comparison with the HCV prototype strain H77 indicating conserved amino acid positions (with red dots) and variable sites (letters).

HCV NS5B gene of 20 isolates were amplified while 13 (65%) of the 20 were successfully sequenced. The sequences have percentage identity of 97%- 100% with reference sequences obtained from GenBank HCV databases. Phylogenetic analysis of the 13 isolates showed three HCV genotypes 1, 3 and 5 (subtypes 1a, 3a and 5a). Ten isolates (77%) were subtype 5a, followed by 2 isolates (15%) subtype 1a and 1 (8%) was subtype 3a. Fig 1 shows the phylogenetic tree of the NS5B gene sequences of the isolates, while Fig 2 shows the amino acid translation indicating conserved and variable sites in the NS5B region. Amino acid substitutions in NS5B gene was compared with the prototype strain H77. Major mutations observed among the genotype 1 isolates (1a) were at positions 61 (G61K) and 89 (G89E). At the other positions, all the amino acids were conserved. In the lone genotype 3, there were substitutions in the following positions: 15 (S15G), 7(T7V), 54 (S54C), 71 (T71S) 89 (G89L) and 80 (T80I). Major mutations among the genotype 5 (5a subtype) isolates were at amino acid positions 89 (G89R) and 84 (S84C).

Two genotype 5 isolates (p9 and p15) generated two variants each. These variants when compared differ by six codons (4 in p9 and 2 in p15), in their nucleotide sequences, representing six different amino acid translations (substitutions) at positions 2, 7, 22, 23, 54 and 81 altogether. At amino acid position 7, P9a had amino acid Thymine (T) while 9b had Serine (S). At position 22, P9a had T while P9b was deleted. At position 23, P9a had amino acid Lysine (L) while P9b had Valine (V). Lastly, at position 81, P9a variant had amino acid Serine while P9b variant had Lysine as its amino acid translation. P15 isolates when compared only had a few substitutions at positions 2 and 54.

## Discussion

This study has shown HCV genotype 5, (subtype 5a), as the predominant HCV strain circulating among HIV/HCV co-infected patients in the study area in Nigeria, with the other genotypes being 1 and 3. Previous studies also showed genotype 5 among HCV- infected blood donors and patients with clinical symptoms [41]. Genotype 5 has not been reported among co-infected patients before now. However, multiple genotypes including 1, 2 and 4 have been previously reported among the general population in Nigeria [42, 43], in addition to numerous reported sero-prevalence studies [44]. These reports show that even within regions in Nigeria, HCV genotypes are differentially distributed and this has serious implication for HCV vaccine development and management of infection in the country. The predominance of genotype 5 among HCV infected individuals co-infected with HIV, may represent a trend of infection with the genotype. However, a larger study may be needed in the population, to provide further information on HCV types and pathogenesis among HIV co-infected population. HCV genotype 5 is not distributed globally [45] and as such, it has not been widely studied in infected persons, making its predominance among this population an interesting information and concern with regards to treatment and management.

Genotype 1 is globally distributed and it accounts for most HCV infections in several parts of the world [46, 47]. In the present study, only 2 subtype 1a isolates (15%) were found among HIV/HCV co-infected patients, contrary to previous studies among blood donors and patients with clinical hepatitis where 8(80%) and 2(20%) of the isolates were subtypes 1a respectively; and 5(100%) were subtype 1b[41]. In Nigeria, limited information exists on HCV types among co-infected patients. There is therefore, the need to carry out further molecular studies to generate additional data that would translate into meaningful policies for the management and survival of HIV/HCV co-infected patients in Nigeria and other parts of the world.

These isolates are distinct considering the significant variation observed at the nucleotide and amino acid levels with bootstrap value of 1000 replicates. Genotype 5 isolates from the study clustered closely with those from South Africa, while genotype 1 clustered with those from Canada and Argentina. The lone genotype 3 isolate clustered with a mutant strain from Sri-Lanka with 97% identity.

Despite availability of direct acting antiviral (DAA) agents, HCV remains a health challenge in developed and developing countries, but worse in the latter, where the supply of these antiviral agents is very limited or not yet approved. It is not clear whether genotype 5a found among HIV /HCV co-infected patients in this study affect the pathogenesis and severity of HIV disease than the other HCV genotypes, as this is beyond the scope of this study. Prior to this study, very little was known about the extent of HCV/HIV co-infection and prevalent genotypes in the study area.

The finding reported in this paper is vital for future policies on vaccine development and antiviral therapy suitable for HCV genotypes in West African region, especially with regards to HCV/HIV co-infection, which facilitates progression to cirrhosis and HCC, and obviously a major public health challenge [48, 49]. Findings from this study update information on HCV diversity among co-infected population. Previously genotypes 1 and 2 were reported some fourteen years ago in Northern Nigeria [50], which is consistent with previous findings in the general population in Africa; but differs from the present study in southwest Nigeria, where genotype 5 and 3 are being reported in addition to the genotypes earlier reported in Nigeria among co-infected individuals. This study, therefore, advances knowledge on the geographical distribution and epidemiology of HCV genotypes and subtypes around the world, a trend with consequent implications for therapy and vaccine design. HCV genotype 5 is quite uncommon in most parts of the world. Its spread might have been facilitated by globalization that continues to impact the epidemiology of HCV globally.

Presently, there is no vaccine for prophylaxis against hepatitis C infection. Hence, additional studies are required to further understand the molecular epidemiology of and immunological factors in infected individuals that elicit interactions between these diverse strains of HCV and their hosts, especially with regards to disease progression in mono-infected and co-infected patients, as well as to determine drug -resistant markers in the diverse HCV strains that may halt or delay response to available antiviral cocktails in Nigeria.

## Conclusion

In conclusion, three HCV genotypes 1, 3 and 5 were found circulating among HIV/HCV co-infected persons in Ibadan, Nigeria, with the predominant strain as genotype 5 S1 Fig. Mutations observed at the NS5B gene of HCV isolates have implications for therapy and monitoring of drug resistance, especially in regions, where the use of DAAs is limited, due to costs of procurement of the drugs. The high prevalence of genotype 5 among HCV patients co-infected with HIV has implication for diagnosis and treatment of this population in Nigeria.

## Supporting information

S1 Fig Distribution of HCV subtypes in HIV/HCV co-infected population. The vertical axis of the graph represents percentage, while the horizontal axis represents HCV subtypes (1a, 3a and 5a found in the study).
(DOCX)
