# Correction: Phylogenetic analysis of hepatitis C virus among HIV/ HCV co-infected patients in Nigeria

**DOI:** 10.1371/journal.pone.0225679

**Published:** 2019-11-19

**Authors:** Juliet A. Shenge, Georgina N. Odaibo, David O. Olaleye

The following information is missing from the Data Availability statement: NS5B sequences have also been deposited to GenBank under accession numbers LC484047-LC484058 and LC506601.
