# Supplementary figures and images for: Phylogenetic analysis of hepatitis C virus among HIV/ HCV co-infected patients in Nigeria
Source: PLoS One. 2019 Feb 6;14(2):e0210724. doi: 10.1371/journal.pone.0210724 (PMC6364902; doi:10.1371/journal.pone.0210724)

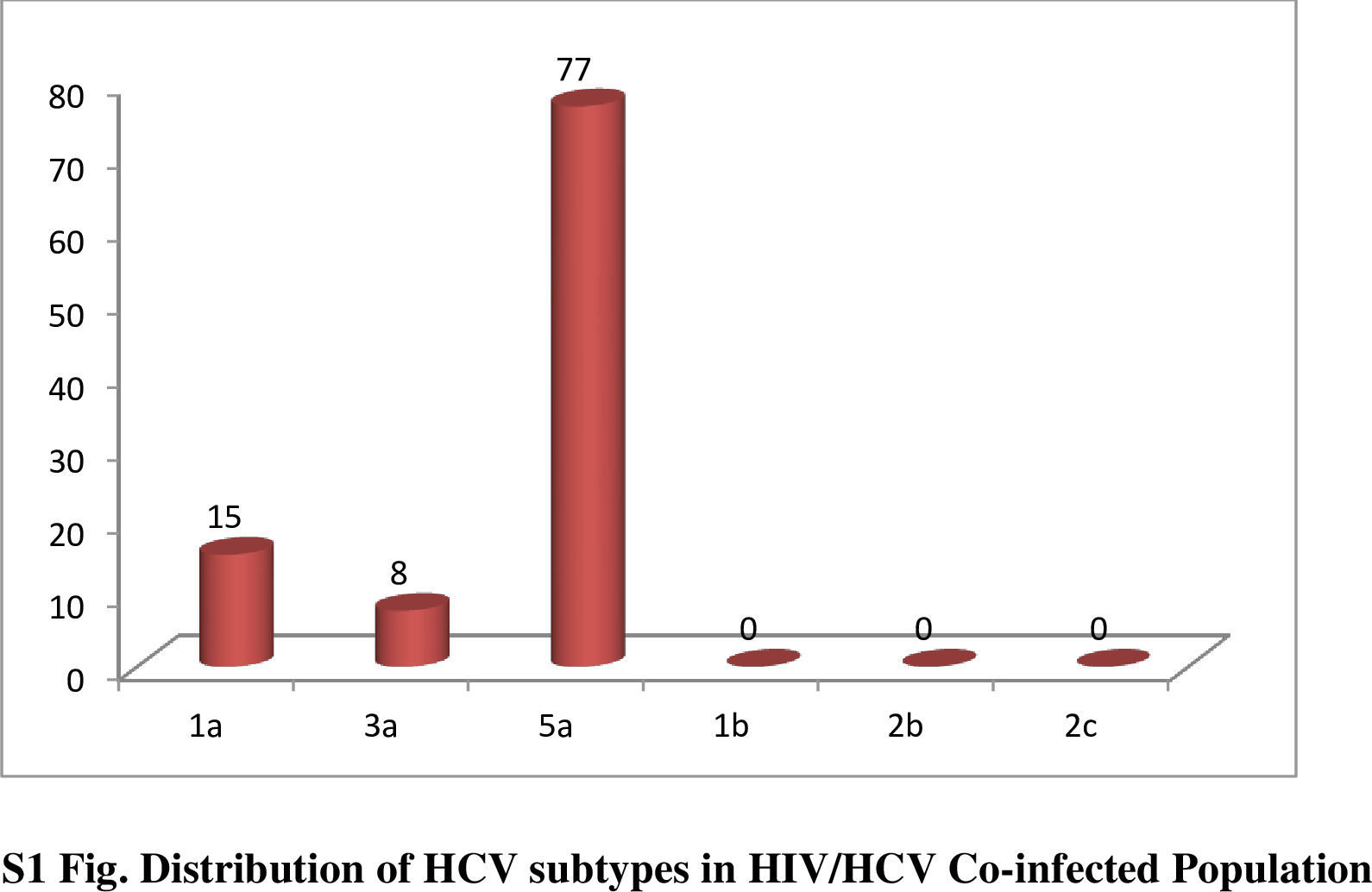

Supplement: S1 Fig — The vertical axis of the graph represents percentage, while the horizontal axis represents HCV subtypes (1a, 3a and 5a found in the study). (DOCX) [file pone.0210724.s001.docx]
